# Discovery of a novel potentially transforming somatic mutation in *CSF2RB* gene in breast cancer

**DOI:** 10.1002/cam4.4106

**Published:** 2021-11-02

**Authors:** Mamoon Rashid, Rizwan Ali, Bader Almuzzaini, Hao Song, Alshaimaa AlHallaj, Al Abdulrahman Abdulkarim, Omar Mohamed Baz, Hajar Al Zahrani, Muhammed Mustafa Sabeena, Wardah Alharbi, Mohamed Hussein, Mohamed Boudjelal

**Affiliations:** ^1^ Department of Bioinformatics King Abdullah International Medical Research Center (KAIMRC) King Saud bin Abdulaziz University for Health Sciences (KSAU‐HS) MNGHA Riyadh Saudi Arabia; ^2^ Medical Research Core Facility and Platforms King Abdullah International Medical Research Center (KAIMRC) King Saud bin Abdulaziz University for Health Sciences (KSAU‐HS) MNGHA Riyadh Saudi Arabia; ^3^ Medical Genomics Research Department King Abdullah International Medical Research Center (KAIMRC) King Saud bin Abdulaziz University for Health Sciences (KSAU‐HS) MNGHA Riyadh Saudi Arabia; ^4^ Research Network of Immunity and Health (RNIH) Beijing Institutes of Life Science Chinese Academy of Sciences Beijing China

**Keywords:** breast cancer, breast cancer cell line, *CSF2RB*‐activating mutation, cytokine receptor, hβc receptor, JAK2 inhibitor

## Abstract

The colony stimulating factor 2 receptor subunit beta (*CSF2RB*) is the common signaling subunit of the cytokine receptors for IL‐3, IL‐5, and GM‐CSF. Several studies have shown that spontaneous and random mutants of *CSF2RB* can lead to ligand independence in vitro. To date, no report(s) have been shown for the presence of potentially transforming and oncogenic *CSF2RB* mutation(s) clinically in cancer patients until the first reported case of a leukemia patient in 2016 harboring a *germline*‐activating mutation (R461C). We combined exome sequencing, pathway analyses, and functional assays to identify novel somatic mutations in KAIMRC1 cells and breast tumor specimen. The patient’s peripheral blood mononuclear cell (PBMC) exome served as a germline control in the identification of somatic mutations. Here, we report the discovery of a novel potentially transforming and oncogenic *somatic* mutation (S230I) in the *CSF2RB* gene of a breast cancer patient and the cell line, KAIMRC1 established from her breast tumor tissue. KAIMRC1 cells are immortalized and shown to survive and proliferate in ligand starvation condition. Immunoblot analysis showed that mutant *CSF2RB* signals through JAK2/STAT and PI3K/mTOR pathways in ligand starvation conditions. Screening a small molecule kinase inhibitor library revealed potent JAK2 inhibitors against KAIMRC1 cells. We, for the first time, identified a somatic, potentially transforming, and oncogenic *CSF2RB* mutation (S230I) in breast cancer patients that seem to be an actionable mutation leading to the development of new therapeutics for breast cancer.

## Graphical abstract

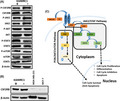

## 1 INTRODUCTION

Over the past quarter of a century, many studies have identified the “driver mutations” offering growth advantage to cancer cells and the genes harboring them “cancer genes” that are causally implicated in cancer development across different types of cancers.^1^, ^2^, ^3^, ^4^ DNA sequencing studies (including WES and WGS) using breast cancer tissues and their patient‐matched normal samples identified several known and novel somatic mutations and copy number aberrations leading to the discovery of “breast cancer genes” and the related “driver mutations.”^5^, ^6^, ^7^, ^8^, ^9^ In 2012, The Cancer Genome Atlas (TCGA) conducted multiple assays, namely genomic DNA copy number arrays, DNA methylation, exome sequencing, messenger RNA arrays, microRNA sequencing, and reverse‐phase protein arrays on primary breast cancer samples and germline DNA samples from 825 patients. This study presented an integrated analysis of multi‐platform data to prove the earlier observation of breast cancer subtypes based on the gene expression profile. Another contemporary study on 103 whole‐exome sequences and 22 whole‐genome sequences of breast cancer/normal pairs confirmed many recurrent somatic mutations in PIK3CA, TP53, AKT1, GATA3, MAP3K1, along with the discovery of recurrent mutations in the transcription factor CBFB and deletion of its partner RUNX1.^5^ Large scale analysis of the 100 breast tumors for somatic copy number alterations and mutations in coding exons of protein‐coding genes revealed several “driver mutations” in new “cancer genes” including AKT2, ARID1B, CASP8, CDKN1B, MAP3K1, MAP3K13, NCOR1, SMARCD1, and TBX3.^7^ Recently, a large‐scale comprehensive study of 360 primary breast tumors and patient‐matched normal samples captured functional regulatory mutations in the genome’s non‐coding regions, especially in the promoter regions.^9^ This study identified significantly mutated promoters of the three genes FOXA1 (a known driver of hormone receptor‐positive breast cancer), RMRP, and NEAT1 (two non‐coding RNA genes). Mutations in the promoter regions of all the above genes affect their respective transcription factors’ bindings, and thus their expression is altered. Large‐scale exome sequencing using tumor‐normal samples from 216 metastatic breast cancer patients identified 12 significantly mutated genes (ESR1, FSIP2, FRAS1, OSBPL3, EDC4, PALB2, IGFN1, and AGRN).^8^ It has been shown that hormone receptor‐positive (HR+)/Her2 negative (Her2−) metastatic breast cancer presented a high prevalence of mutations in the genes related to the mTOR pathway compared to HR+/Her2− early breast cancer counterparts. The above‐mentioned studies contributed to the finding of new “cancer genes” and “driver mutations” in breast cancer and other cancer as well and the development of handy data resources for cancer research like Catalogue of Somatic Mutations in Cancer (COSMIC)^10^, ^11^ and Cancer Gene Census (CGC).^4^

*CSF2RB*, also known as the common beta subunit (hβc) of receptors for GM‐CSF, IL‐3, and IL‐5, has been shown to harboractivating mutations conferring ligand‐independent activation and tumorigenicity to murine hematopoietic cell lines^12^, ^13^, ^14^, ^15^ and primary hematopoietic cells.^16^ The normal cytokine receptor signaling requires a ligand that binds to a ligand‐specific α chain either in a preformed receptor complex with β subunit (in case of GM‐CSF), or to induce receptor dimerization or oligomerization (in case of IL‐3 and IL‐5) followed by the receptor activation and generation of intracellular signals.^17^ The activating mutations in hβc induce receptor oligomerization, receptor activation, and signaling even in the absence of ligand(s) and promote cell survival, proliferation, and differentiation. The activating mutation location may dictate the underlying mechanism of ligand‐independent activation and require cell type‐specific molecules in signaling. Surprisingly, both the transmembrane mutation (such as V449E) and extracellular mutation (such as I374N) of hβc could confer ligand‐independence on factor‐dependent hematopoietic cell line FDC‐P1 but not on CTLL‐2 (IL‐2‐dependent T‐cell line) whereas only V449E could confer ligand‐independence on BAF‐B03 (IL‐3‐dependent subline of the pro‐B cell line Ba/F3).^14^ In a study on murine primary hematopoietic cells, it has been shown that the extracellular mutations in hβc could induce factor‐independence only on neutrophils and monocytes but transmembrane mutation on neutrophil, monocyte, eosinophil, basophil, megakaryocyte, and erythroid lineages.^16^ So far, these activating mutations of *CSF2RB* have only been identified and characterized in vitro in hematopoietic cell lines and primary cells and not been reported in clinical samples.

The first report of a *CSF2RB*‐activating “germline” mutation in leukemia patient confirmed the oncogenic potential of *CSF2RB* mutations in clinical samples.^18^ The reported R461C *CSF2RB* mutation (a rare germline mutation) was found to activate several signaling pathways such as STAT5, PI3K/mTOR, and MEK/ERK pathways constitutively and promote growth and differentiation. Importantly, this mutation was proved to be a targetable/actionable genetic lesion after screening R461C expressing cells against 104 small molecule inhibitor libraries and JAK inhibitors tofacitinib, ruxolitinib, and AZD1480 constituted the top three hits. Though R461C *CSF2RB* was listed in the 1000 genomes database as an SNP (rs371045078) with low allele frequency, it has never been reported in cancer specimens until its first report in leukemia patient.^18^ Moreover, it was not a somatic mutation. To the best of our knowledge, activating *CSF2RB* mutations (germline or somatic) have never been reported in non‐leukemia cancer patients.

Here, we report the discovery of a novel somatic S230I *CSF2RB* mutation (i.e., neither present in 1000 genomes database, Exome Aggregation Consortium nor in the COSMIC database) in a breast cancer patient that tends to be potentially transforming and oncogenic mutation. This is the first report of a *CSF2RB*‐activating somatic mutation in any type of cancer specimen. The breast cancer cell line KAIMRC1^19^ established from the patient’s breast tumor (harboring *CSF2RB* S230I) was able to survive and proliferate. Moreover, the downstream signaling pathways of *CSF2RB* (such as JAK/STAT and PI3K/mTOR) were found constitutively active in KAIMRC1 cells under starvation condition. A small‐molecule kinase inhibitor library screen against KAIMRC1 cells identified JAK2 inhibitor, ruxolitinib, as the best hit. Five different in silico function prediction methods simultaneously predicted S230I *CSF2RB* mutation highly pathogenic and damaging. Protein structure modeling of the mutant hβc suggested that the 230 amino acid locates on the surface of mutant protein and mutant amino acid isoleucine has different size and biochemical properties than wild‐type serine disrupting intramolecular interactions and possibly leading to the ligand‐independent oligomerization of receptor molecules and inducing signaling through JAK/STAT and PI3K/mTOR pathways.

## 2 MATERIALS AND METHODS

Frozen patient tissue samples were used to extract DNA for Sanger sequencing. These samples were obtained during the surgery from women with breast cancer.

### 2.1 Sanger sequencing

DNA was extracted from peripheral blood mononuclear cells (PBMCs), KAIMRC‐1, MCF‐7, and MDA‐MB‐231 cell lines using the Qiagen DNA extraction kit as per the manufacturer’s protocol. DNA concentration was measured by NanoDrop 3300. Polymerase chain reaction (PCR) was performed using the 2X Dream Taq Hot Start Green master. Nine primers were obtained from MACROGEN Company (Ankyrins‐1, Ankyrins‐2, Ankyrins‐3, Ankyrins‐4, *CSF2RB*‐1, *CSF2RB*‐2, *CSF2RA*‐1, *CSF2RA*‐2, and *FGFR1*‐1). DNA was sequenced by Sanger sequencing protocol on 3730XL using the BigDye Terminator v3.1 Cycle sequencing kit used as manufacturer’s protocol (Thermo Fisher Scientific CO).

### 2.2 Exome sequencing experiment

An Ion Torrent adapter‐ligated library was generated following the manufacturer’s protocol (Ion AmpliSeq™ Exome RDY kit PIv3, Rev. A.0; MAN0010084; Thermo Fisher Scientific, Inc.). Briefly, 100 ng high‐quality genomic DNA was used to prepare the Ion AmpliSeq™ Exome capture library. Pooled amplicons were end‐repaired, and Ion Torrent adapters and amplicons were ligated with DNA ligase. Following AMPure bead purification (Beckman Coulter, Inc), the library’s concentration and size were determined using the Applied Biosystems^®^ StepOne™ Real‐Time PCR system and Ion Library TaqMan^®^ Quantitation kit (both from Thermo Fisher Scientific, Inc.).

Sample emulsion PCR, emulsion breaking, and enrichment were performed using the Ion PI™ Hi‐Q™ Chef 200 kit (Thermo Fisher Scientific, Inc.) according to the manufacturer’s instructions. An input concentration of one DNA template copy per ion sphere particles (ISPs) was added to the emulsion PCR master mix and the emulsion was generated using the Ion Chef™ System (Thermo Fisher Scientific, Inc.). Template‐positive ISPs were enriched, sequencing was performed using Ion PI™ Chip kit v3 chips on the Ion Torrent Proton, and barcoding was performed using the Ion DNA Barcoding kit (Thermo Fisher Scientific, Inc.).

### 2.3 Variant calling

Data from the Proton runs were initially processed using Ion Torrent platform‐specific pipeline software, Torrent Suite v4.0 (Thermo Fisher Scientific, Inc.) to generate sequence reads, trim adapter sequences, filter, and remove poor signal‐profile reads. Initial variant calling from the Ion AmpliSeq™ sequencing data was generated using Torrent Suite with a plug‐in “variant caller” program. To eliminate erroneous base calling, three filtering steps were used to generate the final variant calling. The first filter was set at an average depth of total coverage of >50, each variant coverage of >15, and *p *< 0.01.

### 2.4 Preprocessing the VCF files

The Ion Reporter software‐generated variants in a VCF (variant calling format) file. VCF is a generic format to store the genetic variants in a flat file along with several important genetic and statistical parameters and annotations required to understand the quality and impact of the variants. Generally, each line of the VCF file describes the variants present at a specific locus of the genome. Thus, the VCF files output by Ion Reporter might contain multiple variants in the same line that create issues sometimes in downstream analysis. Therefore, multiple variants per line were split into separate lines using “bcftools” so that each line of the VCF file now represents a single variant. Another important preprocessing step is the “left normalization” of indels, which simply means shifting the start position of the variants to the left side of the genome until it is no longer possible to do so. Left normalization provides a unified framework to represent indel variants and thus the comparison of two variant sets becomes possible. Therefore, the left normalization was performed on each VCF file using “bcftools.”

### 2.5 Variant annotation

We used ANNOVAR^20^ to annotate the genetic variants for the KAIMRC1 cell line and the germline control. Annotation operations included gene‐based, region‐based, and filter‐based annotations. The RefSeq gene model was adopted while performing gene‐based annotation.

### 2.6 Western blot analysis

MCF‐7, MDA‐MB‐231, and KAIMRC1 cells were seeded in six‐well plates in complete DMEM for 24 h. Before protein extraction, cells were pre‐incubated with 10% serum‐containing complete DMEM and serum‐free DMEM conditions for another 24 h. Protein concentration was quantified using Bradford assay (Bio‐Rad). The western blotting analysis was performed with Thermo Fisher Scientific mouse monoclonal antibody against mTOR (215Q18; Cat # AHO1232; 1:500), Rabbit polyclonal antibody to Phospho‐mTOR (Ser2448; Cat # 44‐1125G; 1:500), Rabbit monoclonal antibody to JAK2 ABfinity™ (18H11L8; Cat # 702434; 1:500), Rabbit polyclonal antibody to Phopho‐JAK2 (Tyr1007, Tyr1008; Cat # 44‐426G; 1:250), Mouse monoclonal antibody to STAT3 (9D8; Cat # MA1‐13042; 1:1000), Rabbit polyclonal antibody to Phopho‐STAT3 (Ser727; Cat # MA1‐13042; 1:1000), Invitrogen rabbit polyclonal antibody to *CSF2RB* (Cat # PA5‐28000; 1:1000), Rabbit polyclonal antibody to Phospho‐*CSF2RB* (Tyr593; Cat # PA5‐36654; 1:500), Cell Signaling Mouse monoclonal antibody to Akt pan (40D4; Cat # 2920s; 1:2000), Rabbit monoclonal antibody to Phospho‐Akt (Ser473; D9E XP®; Cat # 4060s; 1:1000), and Rabbit polyclonal antibody to Stat5 (3H7; Cat # 9358; 1:1000), and sample loading was examined by probing with mouse monoclonal antibody against beta‐actin loading control (BA3R; Cat # MA5‐15739, Thermo Fisher Scientific; 1:500). Signals were detected using a ChemiDoc MP System (Bio‐Rad) and analyzed on Image Lab software.

### 2.7 Cell proliferation assay

A panel of small molecule kinase inhibitors was utilized to investigate the role of *CSF2RB* protein in cell proliferation. The CellTiter‐Glo assay (Promega) was used according to the manufacturer’s recommendations. Luminescence was measured using the Envision plate reader (Perkin Elmer). Luminescence readings were normalized to averaged DMSO controls and expressed as a relative percentage. Data were analyzed with GraphPad Prism 8 software and the half‐maximal inhibitory concentration (IC_50_) was determined. Error bars denote standard deviation (SD).

### 2.8 Protein structure modeling

The three‐dimensional mutant S230I protein structure was modeled by the Swiss‐Model program^21^ using the hβc receptor wild‐type (WT) structure (PDB: 1GH7).^22^ Hydrophobicity surfaces on modeled protein structure were presented by Chimera.^23^ The GM‐CSF/GMRα/hβc ternary complex (PDB: 4NKQ)^24^ was used for superimposition analysis.

## 3 RESULTS

### 3.1 Quality control analysis

Once the variants are generated from the Ion Reporter software system and preprocessed, we conducted quality control analysis to compile high‐quality variants set for all the downstream analyses.

#### 3.1.1 Assessing the off‐target mutations

The assessment of the mutations presents outside the defined target exonic regions has been performed using the VCF file from Ion Reporter software and the target exome definition file used in the library preparation step for whole‐exome sequencing. None of the mutations were found outside the target region (Table S1). This may be due to the reason that Torrent Variant Caller called only mutations within the target regions.

#### 3.1.2 Ti/Tv ratio, dbSNP concordance, and proportion of variant types

As QC metrics, we checked several parameters (Table S1) to show the quality of the VCF files produced in this study. The transition (*Ti*) to transversion (*Tv*) ratio (*Ti*/*Tv*) for the KAIMRC1 cell line and normal sample computed to be 2.7 and 2.65, respectively. The dbSNP concordance for the KAIMRC1 exome and the normal sample variants was 96.2% and 97.9%, respectively.

### 3.2 Identification of novel somatic variants in the KAIMRC1 cell line

In this study, the patient’s genetic variants from the PBMC sample served as the germline control set for the breast cancer cell line KAIMRC1. The number of variants (SNVs and INDELS) detected in KAIMRC1 and PBMC control was 32917 and 30939. First of all, the variant set common between KAIMRC1 and PBMC (16700) is removed from further analysis, assuming those as germline variations. Thus, the variant set exclusive to KAIMRC1 contained 16217 variants and was subjected to additional filtration. The first filter applied is avsnp150 (dbSNP150 preprocessed and formatted by ANNOVAR software), and 1098 variants passed the filter and contained for subsequent filtration. A series of variants set (COSMIC, ExAC_ALL, GME, esp, gnomAD, clinvar, ICGC, Nci60, 1000 g) were used to filter out the mutations that were present in these sets, and remaining ones were assumed to be novel for the KAIMRC1 cell line. The number of mutations turned out to be 983 in this novel variant set. These mutations have been classified according to the ANNOVAR annotation using gene function filter and found that 582 mutations are exonic, and the rest are intronic, splice, 5UTR, 3UTR, etc.

**FIGURE 1:**
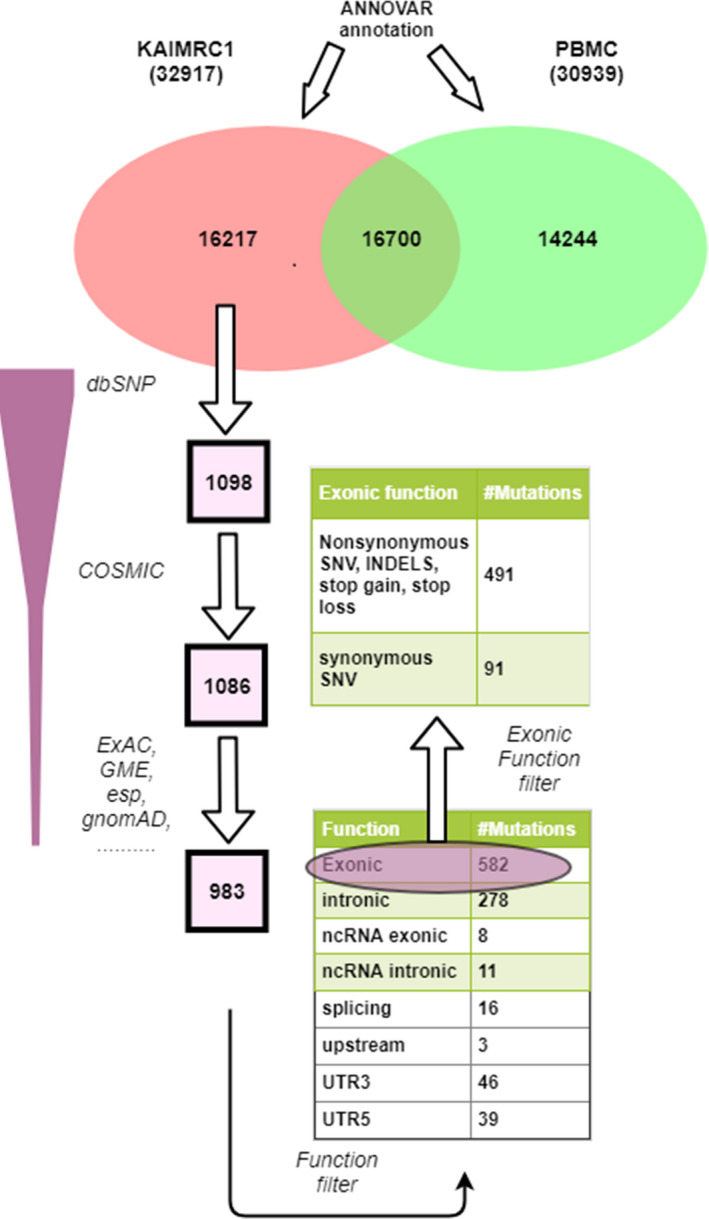
Schematic explaining the exome variants annotation and filtering procedure. Patient’s peripheral blood mononuclear cell (PBMC) was used as a germline control and the variants exclusively present in KAIMRC1 cells followed several filtering steps to obtain novel somatic mutations

Further classification of these 582 mutations for mutation type resulted in 491 mutations (nonsynonymous SNVs, deletions, insertions, stop gain, stop loss) after excluding 91 synonymous SNVs. We proceeded further for downstream analyses with this set of 491 mutations (325 SNVs and 166 others) that are supposed to be novel functional mutations. The whole filtering procedure explained in this section is depicted in Figure 1.

### 3.3 Genes bearing novel somatic mutations and comparison to CGC

Extracting gene IDs for the 491 novel mutations resulted in 423 unique genes. There are a few genes with multiple mutations. Thus, the final number of genes turned out to be 423 harboring novel mutations (SNVs and INDELS) for further analysis. This gene set is compared with the CGC (https://cancer.sanger.ac.uk/cosmic/census?genome=37). CGC is an ongoing effort to catalog those genes which contain mutations that have been causally implicated in cancer. The original study on the “census” of cancer genes indicated that mutations in more than 1% of human genes contribute to cancer.

CGC set containing 723 genes was downloaded from the COSMIC v87 database (date: 23 December 2018). The comparison of CGC and the 423 unique genes from this study resulted in 21 common genes (Table S2). These 21 genes are CGC genes, classified into “Tier 1” and “Tier 2.” Tier 1 genes in CGC are the ones with a well‐documented role in cancer, along with mutations in cancer that promote oncogenic transformation. For Tier 2 genes, there is a strong indication for their role in cancer but with less available evidence. The 21 gene sets are enriched in Tier 1 genes (19/21 = 90.5%), indicating that most of these genes are cancer‐causing genes. In the KAIMRC1 exome, there are novel nonsynonymous mutations in these genes (Table S3). These mutations in CGC genes are missense mutations that may lead to changes in amino acids in the protein product. Since these genetic variations are present in cancer genes, it might explain the possible oncogenic pathways in breast cancer patients.

### 3.4 Pathway analysis of genes with novel mutations in KAIMRC1 cells

The unique gene set (309 genes) was also subjected to pathway analysis using the “Reactome” pathway analysis tool. The pathway enrichment or over‐representation analysis addresses the question, “does the submitted gene list contains more proteins for pathway X than would be expected by chance.” Reactome conducts a binomial test calculating probability value for each result and the p‐values are corrected for the multiple testing (Benjamini–Hochberg procedure). Out of 309, 178 genes were found in Reactome, where 695 pathways were hit by at least one of the genes. The top pathways in the hit are presented in Table 1. A few of the pathways are described below.

**TABLE 1:** The top 10 pathway hits in the reactome analysis of 309‐gene set

Pathway name	Entities				Reactions		Gene names
	Found	Ratio	*p*‐value	FDR	Found	Ratio	
Interaction between L1 and ankyrins	4/33	0.002	0.009	0.615	4/4	3.44e‐04	*SCN11A*, *KCNQ3*, *NRCAM*, *ANK2*
Defective *CSF2RB* causes pulmonary surfactant metabolism dysfunction 5 (SMDP5)	2/8	5.77e‐04	0.017	0.615	1/1	8.59e‐05	*SFTPC*, ***CSF2RB***
Defective CSF2RA causes pulmonary surfactant metabolism dysfunction 4 (SMDP4)	2/8	5.77e‐04	0.017	0.615	1/1	8.59e‐05	*SFTPC*, ***CSF2RB***
Signaling by FGFR1 amplification mutants	2/9	6.49e‐04	0.021	0.615	5/5	4.29e‐04	*FLG*
DAP12 interactions	4/52	0.004	0.04	0.615	8/33	0.003	*VAV3*, *KIR2DS4, CLEC5A*, *VAV2*
Folding of actin by CCT/TriC	2/13	9.37e‐04	0.041	0.615	2/2	1.72e‐04	*CCT6A*
Surfactant metabolism	4/53	0.004	0.042	0.615	11/27	0.002	*SFTPC*, *GATA6*, *CSF2RB*
HDMs demethylate histones	3/32	0.002	0.045	0.615	14/17	0.001	*PHF2*, *KDM2B*, *HIST1H3C*
Regulation of the PAK‐2p34 activity by PS‐GAP/RHG10	1/2	1.44e‐04	0.048	0.615	2/2	1.72e‐04	*PAK2*
Defective pro‐SFTPC causes pulmonary surfactant metabolism dysfunction 2 (SMDP2) and respiratory distress syndrome (RDS)	1/2	1.44e‐04	0.048	0.615	1/1	8.59e‐05	*SFTPC*

#### 3.4.1 Interaction between L1 and ankyrins

Ankyrin family of genes is known to couple the integral membrane proteins (such as L1 CAM, Ion channels) with the underlying spectrin‐actin cytoskeleton. This binding enhances the homophilic adhesive activity of L1 and reduces its mobility within the plasma membrane. L1 interaction with ankyrin mediates branching and synaptogenesis of cortical inhibitory neurons. It has been proposed that ankyrin binding to L1CAMs provides a master switch directing L1‐type proteins into several functional contexts. The genes present in this pathway are SCN11A, KCNQ3, NRCAM, and ANK2 (Table 1).

#### 3.4.2 Defective CSF2RB causes pulmonary surfactant metabolism dysfunction 5 (SMDP5)

*CSF2RB* (colony‐stimulating factor 2 receptor beta) is a high‐affinity common subunit of receptors for GM‐CSF, IL‐3, and IL‐5. Upon ligand binding, granulocyte‐macrophage colony‐stimulating factor receptor (GM‐CSFR), a heterodimer of alpha (CSF2RA) and beta (*CSF2RB*) subunits, initiates a signaling process that not only induces proliferation, differentiation, and functional activation of hematopoietic cells but also perform surfactant uptake by alveolar macrophage and its degradation. The defects in *CSF2RB* are well related to a rare lung disorder called pulmonary surfactant metabolism dysfunction 5 (SMDP5, OMIM: 614370, aka pulmonary alveolar proteinosis 5, PAP5). The genes present in this pathway are SFTPC and *CSF2RB*.

#### 3.4.3 Signaling by FGFR1 amplification mutants

Amplification and activation of fibroblast growth factor receptor 1 (FGFR1) have been reported in many cancers. The gene present in this pathway is FLG.

### 3.5 Validation of mutations by Sanger sequencing

Based on the pathway analysis, few mutations have been selected for validation by the Sanger sequencing technique. From the top four pathways (Table 1), seven unique genes (with seven novel mutations in the KAMRC1 cell line) have been chosen for the dideoxy chain termination sequencing reaction for the validation of the mutant allele. In this assay, the PBMC from the patient (as germline control) and other breast cancer cell lines like MCF‐7 and MDA‐MB‐231 were also included to validate these mutations. Finally, four out of seven (~57%) novel mutations have been validated in KAIMRC1, but they were not present in PBMC, MCF‐7, and MDA‐MB‐231 (Table 2 and Figure S1). Therefore, these four mutations are specific to the KAIMRC1 cell line.

**TABLE 2:** Sanger sequencing of novel somatic mutations in KAIMRC1 cells selected from top four pathways

Chr	Pos	Ref	Alt	Gene model	Sanger sequencing result			
					KAIMRC1^a^	PBMC	MCF‐7	MDAMB‐231
8	133192528	T	C	*KCNQ3*:NM_001204824:exon4:c.A293G:p.Q98R, *KCNQ3*:NM_004519:exon4:c.A653G:p.Q218R	C	T	T	T
7	107880505	G	C	*NRCAM*:NM_001037132:exon1:c.C4G:p.Q2E, *NRCAM*:NM_001193582:exon4:c.C4G:p.Q2E, *NRCAM*:NM_001193583:exon4:c.C4G:p.Q2E, *NRCAM*:NM_001193584:exon4:c.C4G:p.Q2E, *NRCAM*:NM_005010:exon4:c.C4G:p.Q2E	C	G	G	G
3	38921581	T	G	*SCN11A*:NM_014139:exon19:c.A3253C:p.K1085Q, *SCN11A*:NM_001349253:exon23:c.A3253C:p.K1085Q	G	T	T	T
22	37325820	G	T	***CSF2RB*** :NM_000395:exon6:c.G689T:p.S230I	T	G	G	G

a Several passages of the KAIMRC1 cell line including 5, 33, and 60 were tested positive for *CSF2RB* S230I mutation.

### 3.6 Computational assessment of the impact of mutations

The above seven mutations were also subjected to in silico functional analyses to assess their impact. A total of seven different computational algorithms were used for this purpose. Five of them (SIFT, PolyphenHDIV, PolyphenHVAR, Mutation taster, Provean) predicted the impact of mutations (damaging, tolerated, benign, neutral, etc.) on protein function using the mutation information. STRUM and HOPE methods consider protein structure as input and calculate biochemical parameters for wild‐type and mutant amino acid residue. Table S4 shows these computational predictions for all seven mutations. Careful examination showed that *CSF2RB* mutation is predicted deleterious/damaging by all five methods and contributes to sizeable free energy change predicted by the STRUM method that is indicative of destabilizing mutation. The HOPE method concludes that the loss of hydrogen bonding due to *CSF2RB* S230I mutation affects the protein folding (Table S4). Other mutations (KCNQ3, NRCAM, and SCN11A) are also predicted to be deleterious or benign; therefore, they are candidates for further examination.

### 3.7 *CSF2RB* S230I mutation in clinical samples

We have reported the novel somatic mutation *CSF2RB* S230I in one breast cancer cell line named KAIMRC1 and its source tumor tissue. While examining other breast tumors using Sanger sequencing (data not shown), we found 2 (tumors from unrelated individuals) out of 12 tumors (~16%) containing *CSF2RB* S230I mutation. Surprisingly, from both these tumors, we established two breast cancer cell lines; KAIMRC1 and KAIMRC2.^25^ Both these cell lines showed ligand‐independent activation. Nevertheless, we did not find *CSF2RB* S230I mutation in any of the normal breast tissue from breast cancer patients (data not shown). The clinical information of the tumors and patients where this mutation was examined is given in Table S5. No significant clinical difference was observed in tumors with *CSF2RB* S230I mutation compared to those without this mutation.

### 3.8 Activation of downstream signaling factors of *CSF2RB*

**FIGURE 2:**
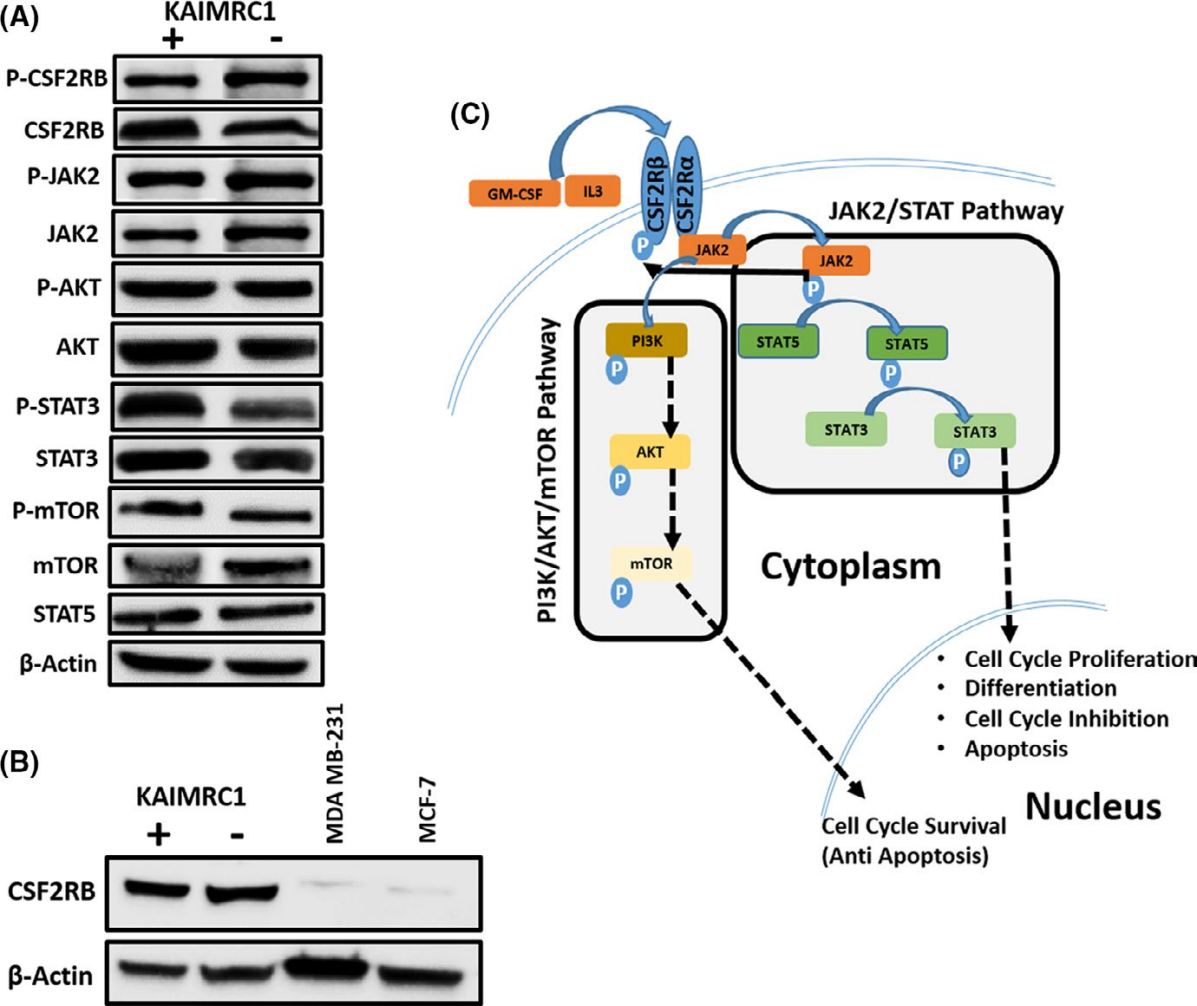
Characterization of KAIMRC1 cells on protein level. (A) Western blot analysis of the KAIMRC1 cell line in normal (+) and serum‐starved conditions(‐) against P‐*CSF2RB*, *CSF2RB*, P‐AKT1, AKT1, JAK2, P‐JAK2, STAT3, P‐STAT3, STAT5, mTOR, and P‐mTOR. Ligand‐independent activation of AKT/mTOR pathway, as well as the JAK2/STAT pathway, was observed. (B) Comparative Western blot analysis of KAIMRC1, MDA‐MB‐231, and MCF‐7 cell lines against *CSF2RB*. (C) Schematic representation of proposed activation of the AKT/mTOR pathway and JAK2/STAT pathway that might result in cell cycle survival and proliferation

Once we know that several downstream kinases of the *CSF2RB* signaling cascade are involved in transformation, we examine their phosphorylation status using specific antibodies. Antibodies against *CSF2RB*, phospho‐*CSF2RB*, JAK2, phospho‐JAK2, STAT3, phospho STAT3, AKT, phospho‐AKT, STAT5, mTOR, phospho‐mTOR are used to screen the KAIMRC1 cells using western blot. KAIMRC1 cells under starvation condition are shown to transduce the cell cycle survival signal through JAK2/STAT and PI3K/AKT/mTOR pathways through *CSF2RB* (Figure 2A). While MDA‐MB‐231 and MCF‐7 cells do not contain the *CSF2RB* S230I mutation, they seem not to signal through the *CSF2RB* receptor (Figure 2B). Figure 2C shows the proposed schematic diagram of signaling events occurring in KAIMRC1 cells through *CSF2RB*.

### 3.9 Small molecule kinase‐inhibitors screening

**FIGURE 3:**
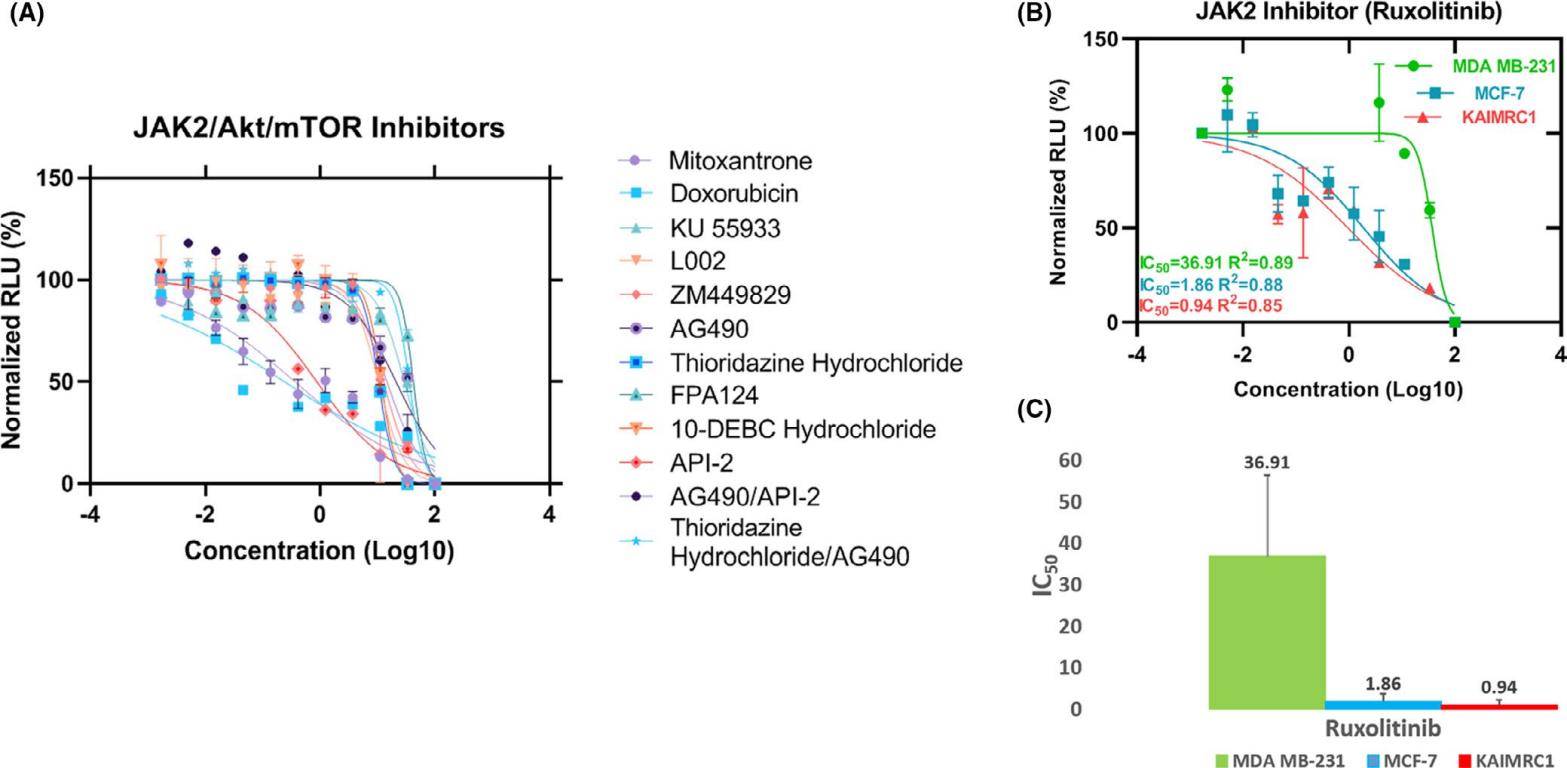
Cell proliferation and Pathway Inhibition Assay. (A) Effect of selected inhibitors of Akt, mTOR, STAT, and JAK on the KAIMRC1 cell line. Akt inhibitor API‐2 showed significant growth inhibition in contrast to other inhibitors, suggesting that the Akt pathway is crucial for the survival of KAIMRC1 cells. (B) Effect of JAK2 inhibitor, ruxolitinib on KAIMRC1, MDA MB‐231, and MCF‐7 cell lines. CellTiter‐Glo® assay was performed to assess cell viability after compound treatment. The cells were treated with compounds in complete media with serum 24 h prior to the assay. (C) Comparison of ruxolitinib IC_50_ of three cell lines. KAIMRC1 cell line showed lowest IC_50_ in comparison to MDA MB‐231 and MCF‐7 cell lines, suggesting that the inhibition of the JAK2 pathway can induce cell cycle inhibition and apoptosis in KAIMRC1 cells. *x*‐axis = Normalized Relative light unit (RLU) and *y*‐axis = Logarithm of the drug concentration in molarity (Log[drug]M)

The role of the novel *CSF2RB* S230I mutation in the transformation of the KAIMRC1 cell line was investigated. A panel of small molecule kinase inhibitors was utilized, specifically, ruxolitinib (a JAK2 inhibitor) and API‐2 (AKT inhibitor), which significantly reduced the growth of the KAIMRC1 cells (Figure 3A and Table 3). Ruxolitinib (JAK2 inhibitor) was found to be very effective in reducing the growth of the KAIMRC1 cells (with *CSF2RB* S230I mutation) in comparison to MDA‐MB‐231 and MCF‐7 cells (without S230I mutation) (Figure 3B,C). It suggests that the growth and survival signaling in KAIMRC1 cells (to a great extent) and MCF‐7 cells (to some extent) are occurring through JAK2 (downstream to *CSF2RB*). Table 3 shows the IC_50_ values of each drug as a percentage. The results indicate the involvement of *CSF2RB* in the ligand‐independent activation of KAIMRC1 cells.

**TABLE 3:** IC_50_ values of the small molecule kinase inhibitors used in this study on the KAIMRC1 cell line

Inhibitors	Category	Pathways	IC_50_
FPA124	Kinase inhibitors	Akt	42.13
10‐DEBC hydrochloride		mTOR/Akt	11.63
API‐2		Akt	0.88
AG490		JAK2/STAT	21.10
ZM449829		STAT5	12.78
Thioridazine hydrochloride	Stem cell modulator	mTOR/Akt/PI3K	10.42
KU55933	Epigenome	mTOR	29.33
L002		STAT3	10.4
Mitoxantrone	Topoisomerase II inhibitor	Antineoplastic agent	0.36
Doxorubicin		Anthracycline	0.24
Ruxolitinib	Kinase inhibitors	JAK1 and JAK2	0.94

### 3.10 Analysis of the hβc receptor S230I mutant protein structure

**FIGURE 4:**
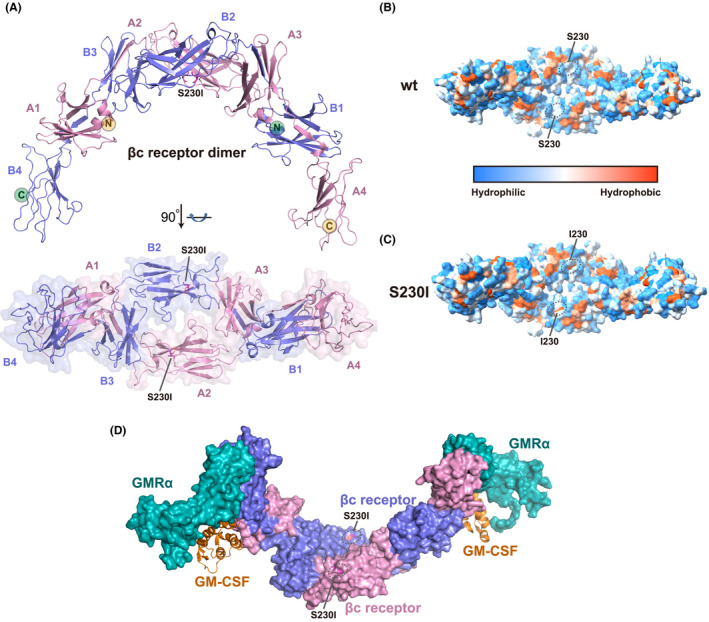
Cytokine βc receptor (CD131) S230I mutant protein structure and analysis. (A) Overall structure of βc receptor (CD131) S230I mutant protein homodimer. The S230I mutant structure was modeled by Swiss‐Model using the wild‐type structure (PDB: 1GH7)^22^ as the template. The S230I residue was colored in magenta and highlighted by ligand shown. Two βc receptor molecules, colored by blue and pink, respectively, form a homodimer. The S230I mutation is located at the second domain of βc receptor. (B,C) Hydrophobic and hydrophilic surfaces of βc receptor wild‐type (B) and S230I mutant (C) protein homodimer. Hydrophobicity surfaces are presented by Chimera^23^ with orange–red for most hydrophobic and dodger blue for most hydrophilic. The residue 230 is highlighted by black ellipses. S230I mutation contributes to more hydrophobic of the βc receptor dimer. (D) Superimposition of βc S230I mutant protein structure on the GM‐CSF/GMRα/βc ternary complex (PDB: 4NKQ).^24^ Both GMRα and βc are shown on the surface. GMRα is colored in teal, and βc dimer is colored as panel A. GM‐CSF is shown in cartoon and colored in orange. The S230I mutation does not involve in the GM‐CSF and GMRα direct binding

Granulocyte‐macrophage colony‐stimulating factor (GM‐CSF) binds and signals through a heterodimeric cell surface receptor complex formed by a specific α subunit (GMRα) and the dimeric common βc subunit (*CSF2RB*). The three‐dimensional structure of the βc receptor (CD131) S230I mutant protein was modeled by the Swiss‐Model program^21^ using the βc receptor wild‐type (WT) structure (PDB: 1GH7)^22^ as the template. The S230I residue is located in the second domain of βc receptor (Figure 4A). Molecular modeling of the mutant protein suggests that the 230 amino acid located on the dimer’s accessible surface and S230I mutation contribute to more hydrophobic of the βc receptor dimer (Figure 4B,C). Although the S230I mutation does not involve GM‐CSF and GMRα direct binding (Figure 4D), this mutation affects the βc receptor stabilization and other functional changes need further study.

## 4 DISCUSSION

Exome sequencing of cancer tissue or primary cells or cell line and paired normal tissue has proven to be a standard method of identifying cancer somatic mutations. Moreover, the integration of genomic and functional analyses yielded cancer driver mutations in the CSF3R gene in leukemia and identified small molecule kinase inhibitors targeting CSF3R downstream signaling pathways.^26^ This manuscript adopted an integrated strategy, including paired‐exome sequencing, pathway enrichment, and functional studies to discover novel somatic mutation *CSF2RB* S230I in a breast cancer cell line KAIMRC1 and related breast tumor. We also found that this mutation leads to the ligand‐independent activation of KAIMRC1 cells through downstream signaling pathways such as JAK/STAT and PI3K/mTOR. Screening the small molecule kinase inhibitor library against the KAIMRC1 cells revealed that JAK2 inhibitor, ruxolitinib, inhibited the growth of KAIMRC1 cells significantly compared to the MDA‐MB‐231 and MCF‐7 cells (without *CSF2RB* S230I mutation). Bioinformatic analyses predicted S230I as a pathogenic variant. In conclusion, *CSF2RB* S230I maybe a transforming and potentially oncogenic mutation in breast tumor.

Several *CSF2RB* mutations, spontaneous or random, have been shown to confer ligand‐independence on hematopoietic cell lines ^13^, ^14^, ^15^, ^27^ or primary cells^16^ in vitro. However, such *CSF2RB* mutations have never been reported in clinical samples until the first case report of a leukemia patient^18^ harboring *CSF2RB*‐activating germline mutation (R461C). R461C mutation was not novel and was present in the 1000 genome database at the time of reporting. Here, we report a similar but completely unknown somatic, potentially transforming, and oncogenic *CSF2RB* S230I mutation in breast cancer tissue and the established cell line KAIMRC1. This mutation has never been reported in any databases such as 1000 genome, Exome Aggregate Consortium, and COSMIC. Nevertheless, few other *CSF2RB* mutations are reported in breast tumors in the TCGA dataset hosted at the COSMIC database (Table S6).

KAIMRC1 cell line, established from a breast tumor specimen, showing growth and proliferation in the ligand starvation condition has been extensively characterized in our lab.^28^, ^29^ We hypothesized that there could be a genetic determinant conferring ligand‐independence to the KAIMRC1 cell line. To answer this question, we designed exome sequencing of the KAIMRC1 cell line and normal PBMC cells obtained from the same breast cancer patient from which KAIMRC1 was established. Using the tumor‐normal exome pair and several publicly available mutation/SNP databases, novel somatic mutations were identified (Figure 1; our bioinformatic framework). The underlying 309 gene set was used for the reactome pathway analysis to identify top pathways where these mutated genes are enriched. The discovery of the *CSF2RB* gene (and its mutation) in the pathway analysis was serendipity and attracted our attention. Since numerous activating mutations were found in the *CSF2RB* gene in hematopoietic cell lines and primary cells (second paragraph of discussion), we hypothesized that S230I in *CSF2RB* might confer ligand‐independence to KAIMRC1 cells. This mutation was not present in MCF‐7 and MDA‐MB‐231 breast cancer cell lines (Table 2) and we considered them (to some extent) as negative controls in our functional studies. Western blot analysis using the monoclonal antibody of *CSF2RB* showed that *CSF2RB* protein was not or least expressed in MCF‐7 and MDA‐MB‐231 cells (Figure 2B) and this further supported their use as negative controls for studying signaling through *CSF2RB* in KAIMRC1 cells.

Surprisingly, MCF‐7 and KAIMRC1 cell lines showed similar IC_50_ of ruxolitinib (a JAK2 inhibitor) treatment (Figure 3C), even though wild‐type *CSF2RB* is not present at a high level in the MCF‐7 cell line (Figure 2B). Ruxolitinib may inhibit MCF‐7 growth and proliferation through a different signaling cascade than the *CSF2RB*/JAK2 pathway. Searching the XTALKDB database,^30^ we found a cross‐talk between the estrogen signaling pathway and JAK/STAT pathway in the MCF‐7 cell line. JAK2 is one of the direct estradiol‐dependent targets of the p/CIP/CARM1 complex.^31^ Therefore, our results imply that in the MCF‐7 cell line, the JAK2 signaling is active through estradiol‐dependent pathways but not through *CSF2RB*.

In silico analysis of *CSF2RB* S230I suggested that “isoleucine” in place of “serine” at 230 could lead to the deleterious or damaging effect on receptor function (Table S4). All the functional prediction bioinformatics algorithms (SIFT, Polyphen2, MutationTaster, Provean) predicted *CSF2RB* S230I as damaging or disease‐causing mutation. Most of these algorithms exploit the sequence conservation level of the amino acid where mutation occurs. The general hypothesis is that the mutation(s) at highly conserved sites might change the structure and function of the respective protein. We sought to confirm whether serine at 230 in *CSF2RB* is highly conserved? Interestingly this site was highly conserved across vertebrates (Figure S2). Moreover, serine and isoleucine differ in size and hydrophobicity; therefore, isoleucine in the mutant *CSF2RB* disrupts the hydrogen bonding with conserved amino acids valine at position 212 and proline at position 231 (Table S4). The disruption of such intramolecular interactions in *CSF2RB* might lead to conformational changes in the receptor finally leading to constitutive activity. Our hypothesis is very strongly supported by a report showing that the *interacting* residues in the extracellular region of the hβc (*CSF2RB*) are involved in constitutive activation.^32^ This report provided mechanistic insight for the *CSF2RB*‐activating I374N mutation. Isoleucine at 374 interacts with leucine at 356 and tryptophan at 358 in the wild‐type hβc. The asparagine (N) at 374 in the mutant receptor disrupts these intramolecular interactions leading to a conformational change in the receptor and leads to constitutive activation. Though we consolidate our hypothesis that mutation at serine 230 (interacting with valine 212 and proline 231) disrupts intramolecular interactions that might lead to receptor activation but more mechanistic studies will be required to support our hypothesis.

S230I *CSF2RB* mutation is not frequent in the breast tumor specimen. We examined 12 tumors and confirmed 2 (16.6%) tumors for this mutation by sanger sequencing. Both these breast tumors gave rise to ligand‐independent cell lines. Our hypothesis, that *CSF2RB* S230I may be an activating mutation in KAIMRC1, was further supported by the extensive reports on hematopoietic cell lines and primary cells where similar mutation(s) in the *CSF2RB* gene confer ligand‐independence and tumorigenicity. The first ever *CSF2RB*‐activating and tumorigenic mutation (but *germline*) in any clinical sample were reported in 2016 in leukemia.^18^ Here, we report for the first time *CSF2RB*‐activating *novel somatic* mutation in breast cancer. This finding could be of translational importance if the anti‐*CSF2RB* antibody could be tested on breast tumors harboring S230I mutation for tumor growth suppression. A similar phenomenon has been observed for CSF1R where humanized anti‐CSF1R monoclonal antibody inhibited both ligand‐dependent and ligand‐independent activation of CSF1R by impairing receptor dimerization.^33^ This manuscript seems to be a foundation stone for exploring *CSF2RB* as a druggable target in the context of breast cancer. Moreover, one can extend this hypothesis to other cancer types as well.

## 5 CONCLUSIONS

*CSF2RB* S230I mutation in breast tumor seems to confer ligand‐independence to breast tumor cells and the KAIMRC1 cell line established from this breast tumor. KAIMRC1 cells harboring this mutation showed constitutive activation of JAK2/STAT and PI3K/mTOR pathways in ligand starvation condition. We found KAIMRC1 cells highly sensitive to JAK2 inhibitors. Together, these findings conclude that *CSF2RB* S230I mutation could be actionable and might help in developing novel therapeutics for breast cancer patients.

## DISCLOSURE STATEMENT

The authors have no conflict of interest.

## ETHICAL STATEMENT

All patients consented following the KAIMRC Institutional Review Board (IRB) guidelines.

## Supporting information

Figure S1. Validation of mutations in genes *KCQN3*, *NRCAM*, *SCN11A*, and *CSF2RB* across breast cancer cell lines using Sanger sequencing protocol.
Figure S2. Conservation of amino acid residues in protein IL3RB_HUMAN.

Table S1. Various quality control metrics for the KAIMRC1 cell line and PBMC normal sample exomes.
Table S2. Genes from Cancer Gene Census present in KAIMRC1 exome with novel mutations.
Table S3. List of novel nonsynonymous somatic mutations in KAIMRC1 exome in Cancer Gene Census (CGC) genes.
Table S4. Computational assessment of the effect of mutations.
Table S5. Clinical information of the breast cancer patients examined for *CSF2RB* S230I mutation by Sanger sequencing
Table S6. Somatic mutations of the *CSF2RB* gene in breast tumor in COSMIC database.

## Data Availability

The datasets generated and/or analyzed during the current study are available in the NCBI SRA repository, (https://www.ncbi.nlm.nih.gov/sra/?term=PRJNA606980).
