# Supplementary material for: Discovery of a novel potentially transforming somatic mutation in CSF2RB gene in breast cancer
Source: Cancer Med. 2021 Nov 2;10(22):8138–50. doi: 10.1002/cam4.4106 (PMC8607246; doi:10.1002/cam4.4106)
Supplement: Supplementary file 1 — Figure S1. Validation of mutations in genes KCQN3, NRCAM, SCN11A, and CSF2RB across breast cancer cell lines using Sanger sequencing protocol. Figure S2. Conservation of amino acid residues in protein IL3RB_HUMAN. [file CAM4-10-8138-s001.pdf]

Supplemental Figures

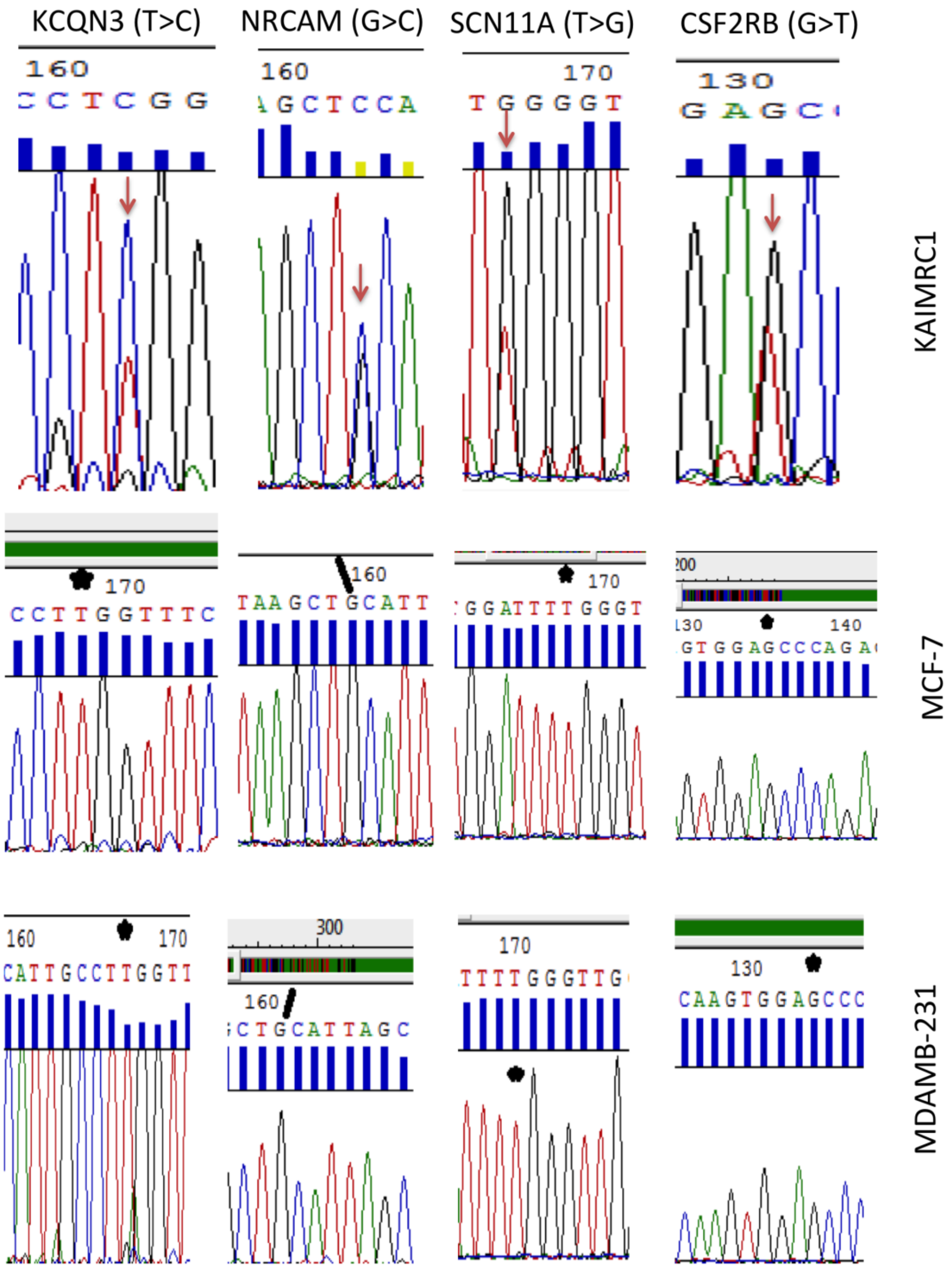

Figure S1: Validation of mutations in genes KCQN3, NRCAM, SCN11A, and CSF2RB across breast cancer cell lines using Sanger sequencing protocol. The rightmost panel shows CSF2RB mutation (G>T) in KAIMRC1 cells but not in MDA-MB-231 and MCF-7 cells. The red arrow and asterisks point site of changes.

mutationassessor.org
functional impact of protein mutations
release 3

cBio@MSKCC

papers | about | privacy | changes | how it works | web API

variant : IL3RB_HUMAN S230I

msa columns order: by specificity submit

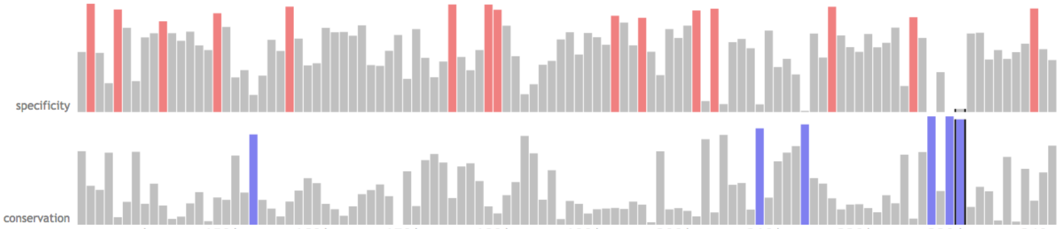


```
                                          |   150|    160|    170|    180|    190|    200|    210|    220|    230|    240
IL3RB_HUMAN/133-240        1  PPEPRDLQISTDQDHFLLTWSVALGSPQSHWLSPGDLEFEVVYKRLQDSWEDAAILLSNTSQATLGPEHLMPSSTYVARVRTRLAPGSRLSGRPSKWSPEVCWDSQPG
tr|F7IKP3|F7IKP3_CALJA     1  PPAPKDLQISTHQDHFLLTWSVALGVPQSQWLSQGDLEFEVVYKRLQDSWEDAASLLSNASQATLGLEHLMPSSTYMARVRTRLASGSRLSGRPSKWSQEFYWNSQPG
tr|F7DHE0|F7DHE0_HORSE     1  PPAPKELQITNARDHFLLTWSMALGDSQSHWLS--NLEFEVVYRRLQDSWEDAPTLLSDSFQAILGPEHLLPSSTYVARVRARLAPGSGLSGRPSQWSPEVRWDSQPG
tr|H0WSU8|H0WSU8_OTOGA     1  PPAPQDLQISAIGEHFLLSWNVTLGGPQPHWLSQEDLEFEVVYRRLQDPWEDAPSLRSNTSQVVLGPSHLMPSSTYVARVRTFLVPGSDFSGRPSEWSPEVRWDSQPG
tr|G1LTB8|G1LTB8_AILME     1  APEPKDLLINTTGDRFLLTWTVALPGPQSHWLS--SLEYEVVYKRLQDSWEEASTLQVNSSQAILEPEHLIPSSTYVARVRTHLAPSSGLSGRPSPWSPEVLWHSQRG
tr|F1SKK0|F1SKK0_PIG       1  PPAPRGLHVSAAQGCFLLSWTVAHEGPQSPWLS--SLEFEVVYRRLQDSWEDAHTIHSGSPQVTLGPEHLLPSSTYVARVRTRLAPGSGLSGRPSHWSPEVHWVSQPG
tr|E2RIM5|E2RIM5_CANFA     1  APAPKDLLIDTTGDRFLLTWSVALPDSQGHWLS--NLEFEVAYKRLQDSWEEASTLHLNSSRAVLGPEHLVPSSTYVARVRTHLASNSGLSGRPSQWSPEVRWHSQTG
tr|G3SXI4|G3SXI4_LOXAF     1  PPEPEDLEVSHAGDGFLLTWRAHLGGLQSRWLSERHLESELVYRRLQDSWEEASTLYSSSSQAVLGPEHLVPSSTYVARVRTRLAPGAGHSGKPSKWSPEVRWDSQPG
tr|Q9Z1A0|Q9Z1A0_CAVPO     1  PPPPQDVQINTSGDQVLLTWSVALEGPHTSWLSQRDLEFEVVYKRLHEPWESASTLHSNSSQAALGPELFLPSSTYVARVRTRLARGSGFSGRPSQWSPEVSWSSQPG
tr|G1TK65|G1TK65_RABIT     1  PPPPTDLQISSRGGGFLLSWSVDLGESQRPWLSREDLEFEVLFRRLHDSWEEATSLLSNSSQAILGPEHLTPNSPYVARVRTRLAPGVAFSGRPSEWSSEIQWDSQSG
tr|F1MXH7|F1MXH7_BOVIN     1  PPTPKDLNITATRESLLLFWSIAHEGFQSHWLS--SLEFEVVYKRLQDSWEDALTIHSKSPQAILELKDLQPSSTYVARVRTRLAPGSGLSGRPSQWSQEVSWNSQPG
tr|G3INB9|G3INB9_CRIGR     1  PPPPKDINISPSGDHFLLKWSVPLGDAWVPWLSQEDLQFEVSYKRLQDSWEDAPSLHTSNLWATLEPKLLLPSNTYVARVRTQLSPGSTLSGKPSRWSPEVQWDSQPG
tr|Q3U2T6|Q3U2T6_MOUSE     1  PPLPKNVSISSSEDRFLLEWSVSLGDAQVSWLSSKDIEFEVAYKRLQDSWEDAYSLHTSKFQVNFEPKLFLPNSIYAARVRTRLSPGSSLSGRPSRWSPEVHWDSQPG
tr|Q78ZF5|Q78ZF5_RAT       1  PPPPKDISISPSGDHFLLKWSVPLGDAQVSLLSQKDIQFEVAYKQLQDSWEDASSLHTCNLWVTLEPKLFLPNSIYVARVRAQLAPGSSLSGRPSGWSPEVHWDSPTE
tr|G3UFG8|G3UFG8_LOXAF     1  PPEPEDLQVSHAGDGFLLTWRAHLGGLQSRWLSERHLESELVYRRLQDSWEEASTLLPVLPKAILRAPCAGPG----------VAPSTGFSGKPSKWSPEVRWDSQPG
tr|F7F5T7|F7F5T7_ORNAN     1  PPAPKQLKVKSIGEGFLLSWNVSTEMP----LQ--DLEFEVLYKRQRDSWEDASSYILNRTQMILN-DTLIPGSTYVARIRTRLAPSSTLSGRPSAWSPEETWQSQAG
tr|G5BAV8|G5BAV8_HETGA     1  SLDKNGYFSFQPDRLLHIQRTVTLAGADRYSYSEGDLEFEVVYKRLHESWENASTLHSSSSQVTLGPELLLPSSTYVARVRTRLAPHSSFSGRPSQWSREVHWDSQPG
tr|H0ZK65|H0ZK65_TAEGU     2  PLPPQNLSVSSMTSDFLLTWKTTDRSQG---LE--NLDYEVTYKREWESWEEAASLLSNTTHCSLSREDLVPGSSYVARVRARPGQASNFSGQYSKWSMEVSWKTPEG
tr|F1NCG4|F1NCG4_CHICK     2  PLPPQNLSIRESSGDFLLTWTSPDGSQGLG----SALEYEVAYKREWESWEKAASLLSNTTRCHLSH--LIPGSRYVARVRARPEQGRGFSGHYSEWSTDVSWETPEG
tr|G1KSA1|G1KSA1_ANOCA     2  -LPPQNLVVNITEDDSFLIWEAGI---RNNWL-ENSLDFEVTYKRFWESWEESSSLVSNASQCLL--QHLVPGNTYVARVRSKPGRTSQFVGHYSDWSSVVIWDTPEG
tr|F1NCG4|F1NCG4_CHICK     2  ---PPSVELMKSQE-YELRWKKH-----SLRYGFIKQKYQVAYKREWESWEKAASLLSNTTHCHL--SHLIPGSRYVARVRAR--PEQDYQGYWSEWSEEFIWET---
tr|G1NJ15|G1NJ15_MELGA     2  PLPPQNLSVRESSGAFLLTWTAPDG-SQG--L-GNALEYEVAYKRDWESWEDSLEMHLYSFSVFIPPLHLIPGSRYIARVRARPEQDRGFSGQYSEWSTDVSWETAEG
tr|F6VPW1|F6VPW1_MONDO     3  PPTPKNLQITKNGT-----------QNHQASS---VLMFEVAYKR-WESWEEAFTKSSSTSHVTFGHDHLIPDNR-----RAKTS-----GERSSPWSSEVSWESQEG
tr|G3WKI4|G3WKI4_SARHA     3  PPAPENLKIMKNGT-----------QSLQEFL---GLKFKVAYKR-WESWEEASTMDSSEAHVLLEYDHLIPNDP-----RAISS-----AGYSSPWSHEFSWNSQEG
tr|F7FJP7|F7FJP7_MONDO     3  PEAPFDLNITYRKADFVLTFSTTHS--WNDYV--KELIHDIAYHQEQSDWT---HIRSSYPHITLLERKLQPNSTYEVKIRSF--PGEYFSGYWSDWSPTVYFRTPL-
tr|H9GEL3|H9GEL3_ANOCA     3  PEAPFNLKITYLEKEYLVDFSSRHG--PKTYLS-GKLMHELAYRQESTPWTNIES--QYTPLKILG-KKLQPGSRYEMRVRSKPH-GGYFEGTWSEWSSS--------
tr|H0ZK68|H0ZK68_TAEGU     4  --------------TSNLTWSLNI---SSHYLD-GRREYQVRYRHVSQSWEEAANLPIEQDQMWANFERLSPNSKYEAAVRARPSARSRYNGIWSDWSKPVLWRT---
tr|F1NDV9|F1NDV9_CHICK     4  --PPEDLQVFSKAKTYNLTWSLNI---SSHYLS-GERQYEVRYRTTSQSWEEAETFIISQDQMWAPFERLSPNMKYEAAVRAKPGASSTCRGVWSDWSKTVLWSTH--
tr|Q6UA82|Q6UA82_CANFA     4  ------------------------------------LEFEARKRSPGHSWEEASLMALRQNQQWISLETLTPDTLYEFQVRVRAQRGS--HKTWSPWSQPLAFRTRP-
tr|F6RXT4|F6RXT4_MONDO     4  ------------------------------------LVFEARYRIQGKPWEEAAVLSIKQDQRWVCIEKLSPDTEYEFQVRGKPERTS--AAVWSPWSQTLVFRTKP-
tr|G3WNS2|G3WNS2_SARHA     4  --ALSNLSLNQQEEAYNVSWEVPV---YSHYL-EGHLIFEVRYQIQGNSWEKATTLPITQDQRWVYLEKLSPNTKYELQVRGK--SKKNGSSGWSPWSPTLIFQTKP-
tr|G1PZN0|G1PZN0_MYOLU     5  --PPSDLQSNVSSDSCVLTWGVSLG--LEPLAS--LLSYELAFKRQEEAWEQGSH--VGVTWLILEAVELDPGSTYEARLRVQMANEERYEGPWSEWSRPVSFLS---
tr|H0VUF3|H0VUF3_CAVPO     5  --PPSDLQSNTSSGSCVLTWSISPP--LEPMAT--LLSYELAFKRQEETWERAQYKDIGVTWIVLEAIELDPGSTYEARLRVQIATEERLEGQWSNWSQSVCFSSP--
tr|G1SXF3|G1SXF3_RABIT     5  --PPSDLQSNISSGSCVLTWSVSLA--LEPLTT--LLSYELAFKRQEEAWEGAQHKDVGVTRITLEAIELEPGSTYEARLRVQMATEERYEGQWSEWSQPVCFRPP--
tr|G5BXZ1|G5BXZ1_HETGA     5  --PPSDLQSNTSSGSCVLTWSISPA--LEPMAA--LLSYELAFKKQEEAWERAQHDIVGVTLLTLEAVELDPGSTYEARLRVQMAMEECHEGQWSKWSQPVCFFSP--
tr|F6PIK9|F6PIK9_HORSE     5  --PPSDLQSNVSSDSCVLTWSITPA--LEPLTS--LLTYELAFKRQEEAWERARHKDVGVTRLILEAVELDPGTTYEARLRVQMATEDRYEGQWSEWSQPVRFPS---
tr|G1L9A1|G1L9A1_AILME     5  --SPSGLQRNISSDYCVLTWSVSPV--VEHLTS--VLSCELAFKEQEEAWEQARHHIVGVTQLTLETVELDPGSHYEARLQVQKATELRCEGQWSEWSQFACFPT---
tr|H2ZZ29|H2ZZ29_LATCH     5  PHPPHNLSVKTTDANFLLAWETEYARDPTGVL-WGAVQYEVSYKRSWETWDDSTFLNEDTPQMELNGLLLDPGVEYSARVRAKPRGDQGFRGHWSEWSPSAMWQSAT-
tr|F1RGX9|F1RGX9_PIG       5  --PPSDVQSNVSSDHCVLTWSVRPA--LEPLAS--LLSYELAFKRQEEAWERARRKDIGVTWLKLEAVELDPGSTYEARLRVQMVTEERYEGQWSEWSLPVRFPAP--
tr|E2QZT2|E2QZT2_CANFA     5  --PPLDLQSNVSSGSCVLTWGVSLA--LEPLAA--ILSYELAFKKQEEAWEQARHRDVGVTRLTLEAVELDPGSSYEARLRVQMATEQHYEGQWSEWSQSVCFPSP--
tr|G3TPN5|G3TPN5_LOXAF     5  --PPSALQSNVSSDYCVLTWSISPA--LEPMAG--LLTYELAFKRQEETWEQARHKDVGVTWLILEAMELDPGSTYEARLRVQMAQEERYEGQWSEWSLPVCFLTP--
tr|F1MTF9|F1MTF9_BOVIN     5  --PPSDLQSNVSSEHCVLTWSVSPA--LEPLAM--LLSYELAFKRQEEAWEWARHKDVGVTWLKLEAAELDPGSAYEARLRVQMAAEERYEGVWSDWSQPACFAAP--
tr|I3M006|I3M006_SPETR     5  --PPSDLHSNISSGSCVLTWTISPA--LEPMTT--LLSYELALKRQEEAWEQARHKDVGVTWLILEAIELDPGSTYEARLRVRMAVEERHEGQWSEWSQSVCFPSP--
tr|H2ZZ29|H2ZZ29_LATCH     5  -PPPFNLSVSELLDNFKVEWKKALPYP------HMQQKYELQFKKQAEAWEFSETLDQDTTDMLLPKMLLDSGSSYVMRVRTKMLKTLSYDGPWSQWSQEAKWETK--
tr|H0WXY0|H0WXY0_OTOGA     5  --PPSDLHSNISSGYCILTWNISPA--LEPMTT--LLSYELAFKRQEEAWEWAQHKDVGVTWLILEAIELDPGFTYEARLRVQMATGALYSGQWSEWSHPVCCSSP--
tr|Q5U4K2|Q5U4K2_XENLA     5  -PPPRWLMSDKLKDTVILSWKSPVPST--FHL---TMLYQIAYRRQWEGWKDSASNVTKELNVSLSPRLFVSGSTYIFRVRTLPHDGEQPKGSWSKWSKELTMKIPEG
tr|G1Q8J5|G1Q8J5_MYOLU     6  --APDSLEVTQEGSTCNVTWTVPQA---SHYL-ERHLEFEVRTRSLDRSWEEASLLKQNQQWIFL--ETLAPDTAYELQVRVRAQRGSLL--TWSPWSQPLAFRTRP-
tr|Q8CBU1|Q8CBU1_MOUSE     6  --APHSLQVLHIDTRCNISWKVSQ---VSHYI-EPYLEFEARRRLLGHSWEDASVLSLKQRQQWLFLEMLIPSTSYEVQVRVK--AQRNNTGTWSPWSQPLTFRTRP-
tr|I3LD35|I3LD35_PIG       6  --PPNSLQVAHIGTRCNITWNLSQ---FSHYL-QSYVEFEVRTRSPGLSWEEGLL--SRGSHCXL--ETLAPDTPYELQVRVR--PQLGHQKAWSPWSGPLL------
tr|I3N1D4|I3N1D4_SPETR     6  --APDSLQVLEVGTTCNVTWRTSL---VSHYI-ESYLEFQVRTRTPGHSWEEAALLSLRQQQQWIWLEQLTPRTPYELQVRVR--PRRGDHTVWSPWSRPLAFRTRP-
tr|F1N409|F1N409_BOVIN     6  --PPHSLQVVRIETRCNITWTVSQ---VSHYI-QNDVEFEARLRYADHSWEDARLLTLRQNQQWISLENLAPGTEYELQVRAK--PRLGSHEVWSHWSQPLAFRTVP-
tr|G9K5M5|G9K5M5_MUSPF     6  --APDLLQITHVGTKCNITWKVPQ---SSHYI-ESYLEFEARTRSPGHSWEEASVLILRQNQQWICLETLTPGTLYEFQVRVR--AHLGSHKAWSPWSQPLAFRTRP-
tr|F1SKK0|F1SKK0_PIG       7  -MAPPTLNLTKDREGYILHWKEE-----KMNYPHIGYIFQVQYKKDADSWEETKTVLQKGHSMSLPP--LEPATRYQARVRVKPDPGS-YNGTWSKWSKEQSWDTE--
tr|G3SXI4|G3SXI4_LOXAF     7  -MDRPTLNVTKDGDSYMLRWETQ-----KQYYGHIRHSFEVQYRKDTDSWEESKTTLENTYNMPLLP--LEPSTRYWARVRVKPSLSS-YNGIWSEWSEELSWDTE--
tr|F1MXH7|F1MXH7_BOVIN     7  -MAPPTLNVTKGRDGYILHWREE-----KMSYSHIACIFQVQYKKEGASWEDTKTDFQNAHTMSLPP--LEPASRYQARVRVKPDPGN-YNGIWSEWSEARSWDTD--
tr|Q3U2T6|Q3U2T6_MOUSE     7  -MEPPTLNLTKNRDSYSLHWETQ-----KMAYSFIEHTFQVQYKKKSDSWEDSKTNLDRAHSMDLSQ--LEPDTSYCARVRVKPISN--YDGIWSKWSEEYTWKTD--
tr|F7DHE0|F7DHE0_HORSE     7  -MPPPKLNVTRSTDGYVLLWKAE-----EMMYKHIGHTFQVQYKKDTASWEDSKTSLQNAHSMSLPW--LEPSTKYQARVRVKPTPGG-YNGVWSEWSEERSWGTE--
tr|Q9Z1A0|Q9Z1A0_CAVPO     7  -MAAPTLNVTKDGDTYSLRWVTE-----KMYYSHIENTFEIQYRTAGDRWENSKTTLKNAHNMPLPP--LEPATTYLARVRVKPSPGGAYNGIWSEWSEEQRWTTD--
tr|G3UFG8|G3UFG8_LOXAF     7  -MGCPTLNVTKDGDSYILHWEAD-----KD----LAPNFEVQYRKDTALWKEAKTALQNTYSLWLSG--LEPSTRYWARVRVKPTPAI-YNGIWSEWSEARSWDTE--
tr|G1LTB8|G1LTB8_AILME     7  -MAQPALNVTKSRDGYTLHWTAK-----EMFYKHIGHTFQVQYKKDAASWEESKTTLQNAHSMSLPP--LEPSTRYWARVRVKPTPSG-YRGIWSEWSEECFWDTE--
tr|E2RIM5|E2RIM5_CANFA     7  -MARPTLNVTKNRDGYTLHWMAK-----EMFYKHIGHTFQVQYKKDTVSWEKSVMKLENAHSMSLPP--LEPSTRYQARVRVKPT-RG-YDGVWSEWSEEHFWDTD--
sp|P32927|IL3RB_HUMAN      7  -MAPPSLNVTKDGDSYSLRWETM-----KMRYEHIDHTFEIQYRKDTATWKDSKTTLQNAHSMALPA--LEPSTRYWARVRVRTSRTG-YNGIWSEWSEARSWDTE--
tr|F7IKP3|F7IKP3_CALJA     7  -FTLSPL----------WGWSGT-----STKMQSRDHNFETEFRSCTNP-QDSKTTLPNAHSMTLPA--LEPSTMYRARVRVKTSRSG-YKGIWSQWSEERSWETE--
tr|Q78ZF5|Q78ZF5_RAT       7  -MNPPTLNLTKNRDSYSLHWETQ-----KMSYPFIQHAFQVQYKKKLDRWEDSKTNLNHAHSMDLPQ--LEPGTSYCARVRVKTIPE--YKGLWSEWSNECTWTTD--
tr|H2TYM5|H2TYM5_TAKRU     8  --------------NALLTWKYPPGDLQYGWI---TLVYELQYRREAENWKVKPS--LWETHLEL--LS-LPVGDYVVRVRCRSK----NSQLWSKWSSAVL------
tr|Q63968|Q63968_9MURI     8  ---------------------------------------RTGYNGIWSEWSEAHS-------MAL--PALEPSTRYWARVRVRTS-RTGYNGIWSEWSEARSWDTE--
tr|G3PSA4|G3PSA4_GASAC     8  --------------AALLSWSYPPSDLRYGWI---TLVYELQYRREPDAWKVKHP--LREPQVEL--LG-LPAGDYVVRVRCRSQ----NSGLWSAWSAAMP------
tr|Q6UAQ2|Q6UAQ2_TETNG     8  --------------NALLSWRYPPGDLQYGWI---TLVYELQYRRDAGNWKVKPS--LREPQVEL--LS-LPVGDYVVRVRCRSK----NSPLWSKWSALMP------
tr|A8WH92|A8WH92_DANRE     8  ---------------SNVSWSKGSNFP----KSIKKHEFQLQFKDAHTSWEMAKPQLSQENYTQLNHDLLTVGEEYQARVRVKPVEDGHFRGEWSEWSPAVSWRSEIG
tr|I3J6V4|I3J6V4_ORENI     8  --------------NALISWTYPPAHVQYGWI---TLVYELQYRREPDNWKVKHP--LREPQVEL--LG-LPVDDYVVRVRCRPQ----HSKLWSDWSATLL------
tr|G4RJE0|G4RJE0_ONCMY     8  ---PGSPVI---INGTNATWSAG--SPLSFKIY--EYEFQVKVKREDQLWGEAQSLTRL------KQEPW--DGIHHIRVRVKPTQ--PPSSHWSEWSTTASWTSE--
tr|I3JPI3|I3JPI3_ORENI     9  PNPPQHVNVTEKDNNWIVKWTEPNKSE------KHGLYYQVLYYRKQD--ENSSTLLDFTSWTLLG-KSLAPLQVYQVKVRSLVVPGSKYEGIPSEWTDPVEWTTNEG
tr|F1R4Q0|F1R4Q0_DANRE     9  PKQPFNLTIRNIDGGFNLTWDV--------------LIYRVRVLS-KSSPEKENILQQNQQSMVILSEKLQPGLQHVADVQVAVNPEW-FPSMWSEWSDSVEWGVE--
tr|G3W556|G3W556_SARHA    10  PRAPRNLIYSQGSDVFFLTWDNNYP-PDHFLIE--ELQYMVEIQKKKDTTT-QFEKIYSSLNLTFLAYHLENKSTYTARVRSKVSD-QTYTGQWSDWSPTVEWF----
tr|H3AGM7|H3AGM7_LATCH    10  PKRPENLTIE---TKMRLSWKNPYD--DGSFLS-DELIFQVHYKKKGDPEKNAIENVSATTEYVIISNDLEPGVNYTAKVRVRS---EEYKTDWSDWSEEVEFHS---
tr|H0XTC8|H0XTC8_OTOGA    10  PRAPGNLTVHTNSDTLLLTWSNPYP-PGTFLHS--ELAYMVNVSREHNSS-DF--RIYNVTVLRITASTLKHGVSYTARVRAW---AESYNSNWSEWSPGIRWL----
tr|Q3U905|Q3U905_MOUSE    10  PLAPDNLTLHTNSDEWLLTWNNLYP-SNNLLYK--DLISMVNISREDNPA-EF--IVYNVPRLSFPINILMSGVYYTARVRVR---SQILTGTWSEWSPSITWY----
```

*** all swissprot (release 2014_07) human gene positions have been pre-computed and are currently available.
*** submission of novel variations for scoring is temporarily unavailable.
server time spent : 0.027387 secs.
peak memory used : 3,481,456

Figure S2: Conservation of amino acid residues in protein IL3RB_HUMAN. S230 is highly conserved across vertebrates. This diagram is the output from "mutationassessor" program.
