# Supplementary material for: Discovery of a novel potentially transforming somatic mutation in CSF2RB gene in breast cancer
Source: Cancer Med. 2021 Nov 2;10(22):8138–50. doi: 10.1002/cam4.4106 (PMC8607246; doi:10.1002/cam4.4106)
Supplement: Supplementary file 2 — Table S1. Various quality control metrics for the KAIMRC1 cell line and PBMC normal sample exomes. Table S2. Genes from Cancer Gene Census present in KAIMRC1 exome with novel mutations. Table S3. List of novel nonsynonymous somatic mutations in KAIMRC1 exome in Cancer Gene Census (CGC) genes. Table S4. Computational assessment of the effect of mutations. Table S5. Clinical information of the breast cancer patients examined for CSF2RB S230I mutation by Sanger sequencing Table S6. Somatic mutations of the CSF2RB gene in breast tumor in COSMIC database. [file CAM4-10-8138-s002.docx]

# Supplemental Tables

**Table S1: Various quality control metrics for KAIMRC1 cell line and PBMC normal sample exomes**.

| Metrics | KAIMRC1 cell line | Normal PBMC |
| --- | --- | --- |
| Total variants | 32917 | 30939 |
| SNPs | 31566 | 29730 |
| INDELS | 1351 | 1209 |
| Ti/Tv | 2.70 (23168/ 8569) | 2.65 (21699/ 8194) |
| Het/Hom | 0.86 (15151/17585) | 1.63 (19140/11683) |
| Nonsynonymous/synonymous SNV | 0.892 (7806 / 8743) | 0.851 (7187 / 8443) |
| Target enrichment | 32917/32917 (100%) | 30939/30939 (100%) |
| dbSNP concordance | 0.962 (31669/32917 | 0.979 (30307/30939) |

**Table S2: Genes from Cancer Gene Census present in KAIMRC1 exome with novel mutations**

| **Gene** | **Name** | **Genome Location (hg19)** | **Tier** | **Somatic** | **Tissue Type^*^** | **Role in Cancer^¶^** | **Mutation Types^$^** |
| --- | --- | --- | --- | --- | --- | --- | --- |
| BCL11A | B-cell CLL/lymphoma 11A | 2:60687539-60780405 | 1 | yes | L | oncogene, fusion | T |
| BLM | Bloom Syndrome | 15:91290623-91358509 | 1 | No | L, E | TSG | Mis, N, F |
| CD209 | CD209 molecule | 19:7807925-7812397 | 2 | yes | E | #N/A | Mis |
| FOXO4 | forkhead box O4 | X:70316379-70321934 | 1 | yes | L, M | oncogene, TSG, fusion | T |
| GNA11 | guanine nucleotide binding protein (G protein), alpha 11 (Gq class) | 19:3094650-3121177 | 1 | yes | E, O | oncogene | Mis |
| GPC3 | glypican 3 | X:132670152-133119476 | 1 | No | O | oncogene, TSG | D, Mis, N, F, S |
| HLA-A | major histocompatibility complex, class I, A | 6:29910331-29913232 | 1 | yes | E | fusion | T |
| KMT2D | lysine (K)-specific methyltransferase 2D | 12:49415563-49449107 | 1 | yes | O, E | oncogene, TSG | N, F, Mis |
| LRP1B | LDL receptor related protein 1B | 2:140990755-142888298 | 1 | yes | E, L | TSG | D, Mis, N, F |
| MDM2 | Mdm2 p53 binding protein homolog | 12:69202258-69233629 | 1 | yes | M, O, E, L | oncogene | A |
| MLLT1 | myeloid/lymphoid or mixed-lineage leukemia (trithorax homolog, Drosophila); translocated to, 1 (ENL) | 19:6213053-6279795 | 1 | yes | L | fusion | T |
| MUC1 | mucin 1, transmembrane | 1:155158611-155162634 | 1 | yes | L | fusion | T |
| MUC16 | mucin 16, cell surface associated | 19:9060452-9091814 | 2 | yes | E | oncogene | Mis |
| NSD1 | nuclear receptor binding SET domain protein 1 | 5:176562105-176722460 | 1 | yes | L | fusion | T |
| PDE4DIP | phosphodiesterase 4D interacting protein (myomegalin) | 1:144852458-144994731 | 1 | yes | L | fusion | T |
| PICALM | phosphatidylinositol binding clathrin assembly protein (CALM) | 11:85670089-85779822 | 1 | yes | L | fusion | T |
| PPM1D | protein phosphatase, Mg2+/Mn2+ dependent 1D | 17:58677776-58740913 | 1 | yes | E | oncogene | A, Mis, N, F |
| PTPRK | protein tyrosine phosphatase, receptor type, K | 6:128291372-128841503 | 1 | yes | E | TSG, fusion | T |
| SDHA | succinate dehydrogenase complex, subunit A, flavoprotein (Fp) | 5:218471-256535 | 1 | yes | M, O | TSG | Mis, N |
| TET2 | tet oncogene family member 2 | 4:106155100-106197676 | 1 | yes | L | TSG | Mis, N, F |
| UBR5 | ubiquitin protein ligase E3 component n-recognin 5 | 8:103266530-103424462 | 1 | yes | L, E | TSG | F, N, Mis, S |

* L: leukaemia/lymphoma, E: epithelial, M: mesenchymal, O: other; ¶ TSG: Tumour Suppressor Gene; $ T: translocation, Mis: missense, N: nonsense, F: frameshift, D: large deletion, S: splice site, A: amplification; this table is complete in Table S5.

**Table S3: List of novel nonsynonymous somatic mutations in KAIMRC1 exome in Cancer Gene Census (CGC) genes**

| CGC Gene | Mutation location (hg19) | Mutation (RefSeq model) | VAF | Mutation Type |
| --- | --- | --- | --- | --- |
| BCL11A | 2: 60689262 | BCL11A:NM_018014:exon4:c.C785T:p.T262I | 0.4615 | SNV |
| BLM | 15: 91326096 | BLM:NM_000057:exon13:c.T2600C:p.L867S | 0.3333 | SNV |
| CD209 | 19: 7810500 | CD209:NM_001144894:exon2:c.G520C:p.E174Q | 0.4285 | SNV |
| FOXO4 | X: 70316613 | FOXO4:NM_005938:exon1:c.236_237del:p.G79Afs*36 | 1 | FD |
| GNA11 | 19: 3110198 | GNA11:NM_002067:exon2:c.A188G:p.H63R | 0.25 | SNV |
| GPC3 | X: 132888138 | GPC3:NM_001164619:exon2:c.C241A:p.P81T | 0.3636 | SNV |
| HLA-A | 6: 29911930 | HLA-A:NM_001242758:exon4:c.651_652TG:p.I218V | 0.9807 | NS |
| KMT2D | 12: 49433117 | KMT2D:NM_003482:exon33:c.C8254T:p.P2752S | 0.2470 | SNV |
| KMT2D | 12: 49445056 | KMT2D:NM_003482:exon10:c.T2410G:p.L804V | 0.2631 | SNV |
| KMT2D | 12: 49445056 | KMT2D:NM_003482:exon10:c.2409_2410insG:p.L804Vfs*8 | 0.5789 | FI |
| LRP1B | 2: 140990763 | LRP1B:NM_018557:exon91:c.G13792A:p.V4598M | 0.6 | SNV |
| MDM2 | 12: 69233496 | MDM2:NM_001145340:exon5:c.G755A:p.G252D | 0.25 | SNV |
| MLLT1 | 19: 6213983 | MLLT1:NM_005934:exon9:c.1373dupC:p.P459Tfs*25 | 0.4461 | FI |
| MUC1 | 1: 155161794 | MUC1:NM_001204285:exon2:c.338_339GC:p.P113R | 0.5909 | NS |
| MUC16 | 19: 9001869 | MUC16:NM_024690:exon53:c.C40379A:p.A13460E | 0.3037 | SNV |
| NSD1 | 5: 176636655 | NSD1:NM_022455:exon5:c.A1255C:p.S419R | 0.4285 | SNV |
| PDE4DIP | 1: 144854597 | PDE4DIP:NM_001350520:exon38:c.7358_7359AG:p.R2453Q | 0.4521 | NS |
| PICALM | 11: 85694912 | PICALM:NM_001008660:exon13:c.1362_1363AT:p.G455W | 0.1818 | NS |
| PPM1D | 17: 58700939 | PPM1D:NM_003620:exon2:c.T530C:p.V177A | 0.5294 | SNV |
| PTPRK | 6: 128841454 | PTPRK:NM_001135648:exon1:c.T50A:p.L17H | 0.2605 | SNV |
| SDHA | 5: 256515 | SDHA:NM_001330758:exon13:c.C1732T:p.P578S | 0.3037 | SNV |
| TET2 | 4: 106196248 | TET2:NM_001127208:exon11:c.4582delC:p.Q1529Sfs*42 | 1 | FD |
| UBR5 | 8: 103341388 | UBR5:NM_001282873:exon11:c.T1256G:p.L419X | 0.2857 | SNV |

* Frameshift mutations; VAF: Variant Allele Frequency from Ion Proton VCF file; SNV: Single Nucleotide Variation; FD: Frameshift deletion; FI: Frameshift insertion; NS: Nonframeshift substitution;

**Table S4: Computational assessment of effect of mutations**

| **Mutation** | **Sanger** | **SIFT** | **Polyphen2 HDIV** | **Polyphen2 HVAR** | **Mutation Taster** | **PROVEAN** | **STRUM (ddG)** |
| --- | --- | --- | --- | --- | --- | --- | --- |
| ANK2: p.R1889G | No | D | P | B | D | N | NA |
| KCNQ3: p.Q98R | Yes | T | D | D | D | D | 1.93 |
| **Hope Server:** The wild-type and mutant amino acids differ in size. The mutant residue is bigger than the wild-type residue. There is a difference in charge between the wild-type (neutral) and mutant (positive) amino acids. The mutation introduces a charge that can cause repulsion between the mutant residue and neighboring residues. The residue is located on the surface of the protein; mutation of this residue can disturb interactions with other molecules or other parts of the protein. | | | | | | | |
| NRCAM: p.Q2E | Yes | D | B | B | N | N | 2.87 |
| **Hope Server:** There is a difference in charge between the wild-type (neutral) and mutant (negative) amino acids. The mutation introduces a charge, which can cause repulsion of ligands or other residues with the same charge. The mutation is located within the signal peptide. This sequence of this peptide is important because it is recognized by other proteins and often cleaved of to generate the mature protein. The new residue that is introduced in the signal peptide differs in its properties from the original one. It is possible that this mutation disturbs recognition of the signal peptide. | | | | | | | |
| SCN11A: p.K1085Q | Yes | T | B | B | N | N | NA |
| CSF2RB: p.S230I | Yes | D | D | D | D | D | 3.53 |
| **Hope Server:** The wild-type and mutant amino acids differ in size. The wild-type residue was buried in the core domain of the protein. The mutant residue is bigger than the wild-type residue and probably will not fit. The mutation introduces an amino acid with different properties, which can disturb this domain and abolish its function. The wild-type residue forms a hydrogen bond with, valine at position 212 and proline at position 231. This size difference between wild-type and mutant residue makes that the new residue is not in the correct position to make the same hydrogen bond as the original wild-type residue did. The hydrophobicity of the wild-type and mutant residue differs. The mutant residue is more hydrophobic than the wild-type residue and this difference in hydrophobicity will affect hydrogen bond formation. The mutation will cause loss of hydrogen bonds in the core of the protein and as a result disturb correct folding. | | | | | | | |
| SFTPC: p.S121F | No | D | P | P | N | N | -0.29 |
| **Hope Server:** The wild-type and mutant amino acids differ in size. The mutant residue is bigger, this might lead to bumps. The hydrophobicity of the wild-type and mutant residue differs. The mutant residue is more hydrophobic than the wild-type residue. The mutation introduces a more hydrophobic residue at this position. This can result in loss of hydrogen bonds and/or disturb correct folding. | | | | | | | |
| FLG: p.S1215G | No | T | B | B | N | N | NA |

Sanger: Validation by Sanger sequencing; SIFT: D=deleterious, T=tolerated; Polyphen2: D=probably damaging, P=possibly damaging, B=benign; Mutation taster: D=disease causing, N=polymorphism; Provean: D=deleterious, N=neutral; NA: the results from STRUM and HOPE could not be achieved for these proteins owing to their large size.

**Table S5: Clinical information of the breast cancer patients examined for CSF2RB S230I mutation by Sanger sequencing**

| **Patient #** | **Age** | **Type** | **Origin** | **Grade** | **Markers** | **Pre Treatment** | **Post Treatment** | **CSF2RB S230I** |
| --- | --- | --- | --- | --- | --- | --- | --- | --- |
| P1 | 60 | Ductal carcinoma | Left breast | SBR grade 1/3 | ER+,PR+, HER2-, Ki-67 10% | None | None | NO |
| P2 | 72 | bilateral breast cancer ( Ductal carcinoma) | Right breast | SBR grade 3/3 | ER+,PR+, HER2-, Ki-67 30%,  E-cad+ | None | None | NO |
| P3 | 57 | Infiltrating ductal carcinoma Bronchitis | Left breast | SBR grade 2/3 | ER+,PR+, HER2-, Ki-67 30%, | Post lobectomy 28 years ago | None | NO |
| P4 | 43 | ductal carcinoma | Left breast | SBR grade 2/3 |  | post neoadjuvant chemotherapy | None | NO |
| P5 | 76 | Infiltrating ductal carcinoma | Right breast | SBR grade 2/3 | ER+,PR+, HER2-, Ki-67 25%, | None | None | NO |
| P6 | 52 | Ductal carcinoma in-situ (DCIS). | Right breast | SBR grade 2/3 | ER+,PR+, HER2-, Ki-67 25%, E-cad+ | tamoxifen then letrozole, with vaginal bleeding | None | NO |
| P7 | 52 | Infiltrating ductal carcinoma | Left breast | SBR grade 2/3 | ER+,PR+, HER2-, Ki-67 15%, | Tamoxifen | None | NO |
| P8 | 46 | Infiltrating ductal carcinoma | Right breast | SBR grade 2/3 | NA | Maintained on tamoxifen. Metasasis found | None | NO |
| P9 KAIMRC1 | 63 | Infiltrating ductal carcinoma | Right breast | SBR grade 2/3 | ER+,PR+, HER2-, Ki-67 10% | Maintained on Letrozole | None | Yes |
| P10 KAIMRC2 | 34 | Infiltrating ductal carcinoma | Right breast | SBR grade 3 | ER-,PR-, HER2+, Ki-67 50% | neoadjuvant chemotherapy, Treated with Epirubicin and Cyclophosphamide (EC) followed by Docetaxel, Herceptin, and Pertuzumab with complete response | Maintained on Herceptin and Pertuzumab.  Developed liver bone metastasis | Yes |

Total number of patients examined was 12 but the clinical data for two patients was not available. Patient 9 and patient 10 are not relatives.

**Table S6: Somatic mutations of CSF2RB gene in breast tumor in COSMIC database**

| S. No. | CDS Mutation | AA Mutation | Legacy Mutation ID | Type |
| --- | --- | --- | --- | --- |
|  | c.145G>A | p.D49N | COSM6568920 | Substitution - Missense |
|  | c.376A>T | p.T126S | COSM7694993 | Substitution - Missense |
|  | c.535C>T | p.Q179* | COSM7672934 | Substitution - Nonsense |
|  | c.631C>T | p.R211* | COSM444946 | Substitution - Nonsense |
|  | c.961G>A | p.V321I | COSM7703399 | Substitution - Missense |
|  | c.1027C>T | p.P343S | COSM4776841 | Substitution - Missense |
|  | c.2614C>A | p.Q872K | COSM444947 | Substitution - Missense |

COSMIC search was performed on 06^th^ September 2020 with genome build GRCh37 COSMIC v92 with filters “tissue”=”breast”, “somatic status”= “confirmed somatic” ,‘’sample type”=”tumor sample”, and excluding synonymous mutation.
